# The role of visfatin levels in gingival crevicular fluid as a potential biomarker in the relationship between obesity and periodontal disease

**DOI:** 10.1590/1678-7757-2018-0365

**Published:** 2019-07-29

**Authors:** Deniz ÇETİNER, Ahu URAZ, Seniha ÖZTOPRAK, Gülçin AKÇA

**Affiliations:** 1 Gazi University, Faculty of Dentistry, Department of Periodontology, Ankara, Turkey.; 2 Gazi University, Faculty of Dentistry, Department of Microbiology, Ankara, Turkey.

**Keywords:** Visfatin, Chronic periodontitis, Obesity, Interleukin-6, Tumor necrosis factor alpha

## Abstract

**Objectives:**

Visfatin is an adipokine that plays an important role in immune functions as a growth factor, enzyme, and pro-inflammatory mediator. We aimed to determine the levels of visfatin, interleukin-6 (IL-6) and tumor necrosis factor-alpha (TNF-α) in gingival crevicular fluid (GCF) in both obese/non-obese patients, with/without generalized chronic periodontitis (GCP).

**Methodology:**

Patients were categorized as obese (O) (n=31) or non-obese (nO) (n=19). Groups were divided into four subgroups according to periodontal conditions: (1) periodontally healthy without obesity (nO-Ctrl); (2) GCP without obesity (nO-CP); (3) periodontally healthy with obesity (O-Ctrl); and (4) GCP with obesity (O-CP). Demographic variables, anthropometric and laboratory data were recorded. Periodontal parameters were measured at baseline and 3^rd^ months after either non-surgical periodontal treatment or calorie -restricted diet therapy. At the same time, GCF samples were taken from patients to analyze TNF-alpha, IL-6,and visfatin levels.

**Results:**

Periodontal parameters were significantly higher in the O group than in the nO group (P<0.05). IL-6 levels were higher in the O group than in the nO group (P<0.001). The visfatin levels of the obese patients were reduceddecreased following the treatments (P<0.05). Cholesterol levels were higher in the O group than in the nO groups (P<0.05). IL-6 levels were higher in O-CP and O-Ctrl groups than in the nO-Ctrl group (P<0.05). Compared to the other groups, visfatin levels were significantly higher in the O-CP group but decreased following treatment (P<0.05).

**Conclusions:**

Our findings suggest that visfatin and IL-6 levels in GCF are associated with the pathogenesis of obesity and periodontitis. Within the limits of this study, we considered that there might be an association between the lipid profile and periodontitis on systemically healthy individuals.

## Introduction

Overweight and obesity are defined as the accumulation of fat in body tissues that might impair overall health.^1^ Adults are considered overweight if their body mass index [BMI, calculated as (weight in kg)/(height in meters)^2^] is ≥25 and obese if BMI≥30 kg/m^2^. The prevalence of overweight and obesity has increased worldwide during recent decades.^1^

Obesity is usually related to a chronic low-grade systemic inflammation resulting in significant changes in the concentrations of cytokines and hormones that subsequently leads to the development of obesity-linked disorders, including insulin resistance, type 2 diabetes, cardiovascular diseases, dyslipidemia, and metabolic syndrome.^1^ Since the host response is among the most crucial factors affecting the pathogenesis of periodontal disease, multiple studies have addressed the possible associations between BMI, overweight, obesity, diabetes, the serum level of lipids, cholesterol, and periodontal breakdown, with mixed results.^1,2^ Many studies have demonstrated a positive association between obesity and periodontitis and suggested that obesity-related inflammation might promote periodontitis by secretion of inflammatory markers by the adipose tissue, which might subsequently increase gingival inflammation.^2,3^ The association between obesity and periodontal disease is based on the amassing of white adipose tissue (WAT) and increased secretion levels of adipokines from WAT. WAT is an energy storage organ with some metabolic activities, participating in the endocrine and secretory systems.^4^ It secretes several immune-modulator adipokine molecules, such as adiponectin, leptin, visfatin, resistin, chemerin, tumor necrosis factor-alpha (TNF-α), interleukin-1β (IL-1β), and IL-6.^4^ It has been found that these molecules are involved in a wide range of physiologic and pathological processes, including immunity and inflammation.^5^ Thus, cytokines and hormones released from adipose tissue might play a role in the destruction of periodontal tissue by inducing hyper-inflammatory responses.

Visfatin is identified as a new adipokine, which is involved in the early development of the B-cell growth factor and cytokine-like effects.^6^ Visfatin is a 52-kDa protein that increases pre-B-cell colony release from lymphocytes.^6^ It is a multi-potential mediator that functions as a growth factor, cytokine, an enzyme with a role in energy metabolism, and as a pro-inflammatory mediator.^6^ Visfatin is mainly released from adipose tissue, especially by macrophages, and can also be released from lymphocytes, dendritic, muscle, periodontal ligament and bone marrow cells.^7,8^ Visfatin has an important role in the regulation of immune response. Visfatin inhibits neutrophil apoptosis during infection and inflammation, and increases TNF-α, IL-1β, and IL-6 levels.^6^ Visfatin is also known as nicotinamide phosphoribosyltransferase.^6^ The expression of visfatin is increased under inflammatory conditions, such as rheumatoid arthritis, cardiovascular diseases, type 2 diabetes mellitus, and periodontal disease.^9^ Several studies have investigated the link between periodontitis and obesity, and these investigations have also shown that obesity and periodontitis are also related to gingival crevicular fluid (GCF) levels of adipokines.^2-4,9^ Increased adipocyte levels, such as from visfatin, cause secretion of cytokines, which are known to play an important role in periodontitis and might trigger periodontitis formation and development.^9^

Therefore, the main objective of this study was to analyze the levels of visfatin, IL-6, and TNF-α in obese and non-obese individuals, with or without generalized chronic periodontitis (GCP). Secondarily, we aimed to evaluate metabolic and clinical periodontal parameters, as well as to clarify the relationship between these parameters and adipocytokines. The hypothesis is that adipocytokine molecules are involved in the pathogenesis of inflammatory diseases; if true, individuals who are obese with periodontitis would present increased levels of visfatin, IL-6, and TNF-α in their GCF.

## Methodology

### Study population

From October 2014 to January 2015, the 195 patients diagnosed with obesity in the outpatient clinics of the Endocrinology and Metabolic Diseases Department from Gazi University were potential candidates, being interviewed according to our case definition and invited to be included in this study. The study was conducted in full accordance with applicable ethical principles, including the World Medical Association Declaration of Helsinki, and was independently approved by the Ethics Committee of the School of Dentistry, Ankara University (Protocol 2011.5.1/1). The trial is registered at the domain ClinicalTrials.gov as NCT03470987.

Thirty-one of 195 volunteers accepted and signed the informed study protocol and took part in the study. All individuals were thoroughly informed of the nature, potential risks and benefits of their participation in the study before providing their informed consent. During the same period, 100 consecutive non-obese patients were screened from the Department of Periodontology, School of Dentistry, Gazi University, who were systemically healthy and fulfilled the inclusion criteria, of which 19 agreed to participate.

Fifty individuals (mean age, 41.58±11.5 years; range, 20–68 years), who were obese (O) (n=31, BMI≥30 kg/m^2^) and non-obese (nO) (n=19, BMI<25 kg/m^2^) and with and without GCP were classified into four groups based on the periodontal and anthropometric measurements: (1) nO-Ctrl (n=10), individuals of normal weight with no history of periodontitis and no sites with pocket depth (PD) or clinical attachment level (CAL) >3 mm; (2) nO-CP (n=9), individuals who are normal weight GCP, presenting ≥30% of the sites with bone loss, and at least two non-adjacent teeth with ≥1 sites with PD≥5 mm and CAL≥5 mm in each quadrant, and positive bleeding on probing (BOP); (3) O-Ctrl (n=10), individuals who are obese with no history of periodontitis and no sites with PD and CAL>3 mm; and (4) O-CP (n=21), individuals who are obese with GCP, presenting ≥30% of the sites with bone loss, and at least two non-adjacent teeth with ≥1 sites with PD≥5 mm and CAL≥5 mm in each quadrant, and positive BOP.

### Inclusion and exclusion criteria

Inclusion criteria for all patients in both groups were: having >22 natural teeth; no systemic diseases; being cooperative; having BMI>30 with waist circumference (WC)>88 cm for females and >102 cm for males for obese patients; and >20-years-old.

The following exclusion criteria were considered: localized chronic periodontitis; receiving periodontal therapy/surgery in the last 6 months; pregnancy or use of any hormone therapy; history of antibiotic or anti-inflammatory drug therapy within the last 6 months; current and former smoker; lactating; aggressive periodontitis; and periapical pathologies.

At the beginning of the study, detailed anamnesis was taken from all participants, and age, gender, oral hygiene habits, and any systemic conditions were recorded for each patient.

### Obesity definition

Obesity was evaluated using weight (kg), height (m), and WC (cm) measurements. The same trained examiner performed all measurements, which were taken at 9 a.m. BMI was calculated as weight in kg/height in meters^2^as an indicator of overall obesity. BMI was categorized according to the World Health Organization (WHO) classification: normal weight was identified as BMI<25 kg/m^2^ and obese as >30 kg/m^2^. WC was also measured. This was divided into two categories, normal and high, using the cut-off point of >88 cm for female and >102 cm for male [The National Cholesterol Education Program Adult Treatment Panel III (NCEP ATP-III) guidelines].

Serum triglyceride (TRG)≥150 mg/dL, high-density lipoprotein cholesterol (HDLc)<50 mg/dL for women and <40 mg/dL for men, low-density lipoprotein cholesterol (LDLc)≥130 mg/dL, and total cholesterol ≥200 mg/dL were measured for definition of dyslipidemia.

A dietitian gave calorie-restricted diets to only obese individuals. Patients were followed-up once per week for 12 weeks. All anthropometric measurements and laboratory data were recorded at baseline and 3 months after diet therapy.

### Clinical examination

**Figure 1:**
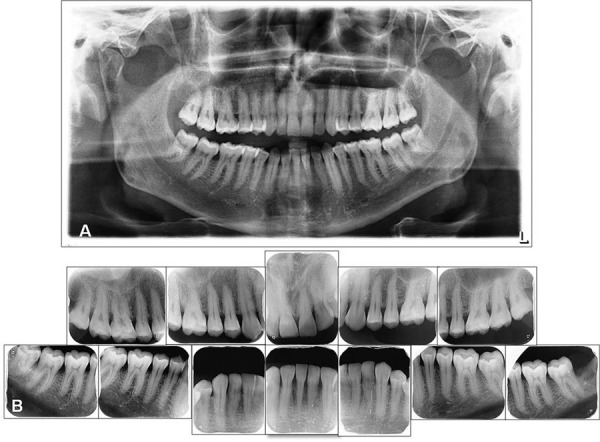
A) Panoramic radiograph of a patient with chronic periodontitis (nO-CP); B) Full mouth periapical radiographs of a patient with chronic periodontitis (nO-CP)

Clinical parameters were measured for midbuccal and midlingual sites as well as the buccal aspects of the interproximal contact area for mesial and distal sites on each tooth using a William’s periodontal probe (Hu-Friedy, Chicago, IL, USA) calibrated in millimeters by a single investigator, who was blind to the research objective and study population at baseline, 3 months after both non-surgical periodontal treatment and diet therapy. Before making the measurements, ten individuals were chosen at random for calibration. These individuals were assessed twice within a 48-h interval. Calibration was accepted if measurements at baseline and 48 hours later did not differ by more than 10%.^4^ The following measurements were recorded for all teeth, excluding third molars; PI (Silness and Löe scale), GI (Silness and Löe scale), BOP (Ainamo & Bay scale), PD, and CAL. Periodontally healthy individuals were those with GI=0, PD and CAL<3 mm, and no attachment/bone loss ascertained through clinical/radiographic examination. GCP patients had clinical signs of inflammation, GI>2, PD and CAL>5 mm, and bone loss affecting >30% of the existing teeth on clinical/radiographic examination. Additionally, periapical and panoramic radiographs were obtained from all patients to confirm the clinical diagnosis and alveolar bone loss (Figure 1).

### Non-surgical periodontal treatment

Once GCF samples were taken, all individuals with GCP underwent full-mouth scaling and root planning (SRP) using hand instruments (Hu-Friedy Mfg. Co., Chicago, IL, USA) under local anesthesia. Non-surgical periodontal treatment was completed in 2-3 visits for 2 weeks based on each patient’s requisites, and each visit was 45–60 min long. In all groups, participants were given standard oral hygiene instructions, using the modified Bass technique, toothpaste, and interdental tools. Pre- or post-operative antibiotics were not prescribed in any group.

### GCF sampling and laboratory analysis

GCF samples were collected 1 week after the clinical measurements taken at baseline and repeated 3 months later. In the healthy group, samples were obtained from maxillary anterior four teeth exhibiting PD<3 mm without CAL or BOP. Four sites from each tooth were used for GCF sampling. In the GCP group, GCF samples were collected from four teeth with BOP, PD≥5 mm, CAL≥5 mm, and 30% bone loss. Before sampling, the area was isolated with cotton rolls to prevent saliva contamination; the area was then slightly air-dried. GCF was collected using paper strips (Periopaper, ProFlow Inc., Amityville, NY, USA) and the volume of fluid in each strip was determined using a calibrated Periotron 6000 (Periotron™ 6000, Proflow Inc., Amityville, NY, USA). Strips were inserted into the crevice until mild resistance was felt, and were put to one side in stasis for 30 seconds. Strips contaminated with blood or saliva were discarded. Samples were immediately placed into microcentrifuge tubes and stored at -20°C until analyzed. Levels of visfatin (Phoneix Pharmaceuticals Inc., Burlingame, CA, USA), TNF-α (eBioscience Inc., Affymetrix, Santa Clara, CA, USA), and IL-6 (eBioscience Inc., Affymetrix, Santa Clara, CA, USA) in GCF samples were determined using an enzyme-linked immunosorbent assay (ELISA) (ELx800, Biotek, Winooski, VT, USA). For laboratory analysis, 600 μl of phosphate buffered saline (PBS, pH 7.4) was added to each tube containing sample strips. Each tube was vortex-mixed for 60 seconds and centrifuged for 5 minutes at 8000 rpm in order to elute. The assay was performed using recombinant human standards according to the manufacturer’s instructions. Optical density was measured at 450 to 490 nm. GCF results were reported as the total amount (pg/ml).

### Statistical analyses

Statistical analysis was performed using commercially available software (SPSS 20.0, SPSS Inc., Chicago, IL, USA). Power analysis was based on the supposition that a mean difference of 0.5 mm in PD should be detected at a significance level of 0.05 and a desired study power of at least 80%. It was estimated that a sample size of at least 20 individuals in each obese and non-obese group would achieve 95% power with a 0.05 two-sided significance level. The required sample size power analysis results into 33 individuals total. In this case, 80.001% of the power test is expected to be obtained. Data were examined for normality by the Shapiro-Wilk test. Continuous variables (age, PI, GI, PD, CAL, and BMI) were presented as the mean±standard deviation (SD). Categorical variables (gender, tooth brushing frequency and interdental cleaning) were expressed in actual numbers. Performing Pearson’s Chi-squared test compared categorical variables. For small numbers, Fischer’s exact test was used. Inter-group comparisons were analyzed by the non-parametric Kruskal-Wallis test, after which the Mann-Whitney U test was utilized for *post-hoc* comparison. The Wilcoxon signed-rank test was performed to analyze intra-group comparisons. The Spearman’s rank correlation coefficient was used to detect the relationship between variables. A *p*-value<0.05 was considered statistically significant.

## Results

Initial demographic data, anthropometric measurements, lipid values, periodontal parameters, and GCF cytokine levels were first evaluated in the O and nO groups, and then in the O-Ctrl group.

### Comparison of data between obese and non-obese groups

Individuals from the O group were older than in the nO group (*P*<0.002). Most of the O group (98%) was female, whereas around half (52.6%) of the nO group was female (*p*<0.001). Additionally, there was a statistically significant difference between groups in terms of tooth brushing frequency (*p*<0.036) and interproximal cleaning (*p*<0.007) (Table 1).

**Table 1:** Demographic characteristics of Obese (O) and Non-Obese (nO) Groups

	O (n=31)	nO (n=19)	P value^**a**^
Age (years) (mean±SD)	45.26±11.1	35.58±9.8	0.002*
Female/male (n)	30/1	10/9	0.001*
Smoking (yes/no) (n)	7/24	5/14	1.000
Frequency of toothbrushing (n) (%)			0.036*
Once a day	19 (61.3)	7 (36.8)	
Twice a day	9 (29.0)	12 (63.2)	
Seldom	3 (9.7)	0 (0.0)	
Interdental cleaning (n) (%)			0.007*
Regular	0 (0.0)	5 (26.3)	
Rare	1 (3.2)	1 (5.3)	
None	30 (96.8)	13 (68.4)	

^a^Comparison of age, female/male, smoking, frequency of toothbrushing and interdental cleaning percentages between O and nO groups by Chi-square test, Ficher’s exact test

*P<0.05, Chi-square test, Ficher’s exact test

SD: Standard deviation

There were differences betwen groups for anthropometric measurements (*p*<0.002). At baseline and 3^rd^ month, weight, BMI and WC were higher in the O group than in the nO group (*p*<0.001) (Table 2). The O group had higher total cholesterol (*p*<0.029) and triglyceride levels than the nO group (*p*<0.05) (Table 2). However, comparing the O and nO groups, no significant differences were detected in any of the laboratory data at the 3^rd^ month (*p*>0.05). At baseline, PI, GI, PD, and CAL values were higher in the O group than in the nO group (*p*<0.05) (Table 2). Only GI scores were statistically different between the O and nO groups at 3^rd^ month (*p*<0.043). Comparing post-therapy and baseline data in the groups, PI, GI, and BOP parameters were reduced following treatment (*p*<0.001). IL-6 levels were higher in the O group than in the nO group (*p*<0.001), whereas TNF-α and visfatin values were similar in both groups at baseline (*p*>0.05) (Table 2). At the 3^rd^ month, there were no statistically significant differences within or between groups for GCF levels of IL-6 and TNF-α. Compared to baseline, there was a significant decrease in the visfatin levels of the O group (*p*<0.05).

**Table 2:** Anthropometric, laboratory, periodontal parameters and total amounts of adipocytokines in GCF of O and nO Groups

	O (n=31)	Between Groups p-values^**a**^	nO (n=19)
Weight (kg)			
Baseline	90.81±13.3	p<0.001*	67.05±11.6
Within the group	N.S		N.S
Month 3	89.33±13.2	p>0.001*	66.84±11.3
Height (m)			
Baseline	161.65±7.0	p<0.001*	167.79±6.9
Within the group	N.S		N.S
Month 3	161.65±7.0	p<0.001*	161.65±7.0
BMI (kg/m2)			
Baseline	34.76±5.6	p<0.001*	23.53±2.8
Within the group	N.S		N.S
Month 3	34.05±5.4	p<0.001*	23.44±2.7
WC (cm)			
Baseline	105.81±10.6	p<0.001*	78.21±12.5
Within the group	N.S		N.S
Month 3	104.65±10.2	p<0.001*	78.21±12.5
Total Cholesterol (mg/dl)			
Baseline	204.48±32.5	p<0.05*	184.26±28.3
Within the group	N.S		N.S
Month 3	195±39.0	N.S	181.44±39.9
Triglyceride(mg/dl)			
Baseline	133.68±46.9	p<0.05*	110.68±61.6
Within the group	N.S		N.S
Month 3	131.71±49.5	N.S	112.36±60.4
HDL (mg/dl)			
Baseline	52.03±12.4	N.S	55.37±15.4
Within the group	N.S		N.S
Month 3	53.77±14.3	N.S	56.21±15.6
LDL (mg/dl)			
Baseline	124.63±27.2	N.S	110.11±28.6
Within the group	N.S		N.S
Month 3	114.68±37.0	N.S	112.89±33.1
PI			
Baseline	0.81±0.50	p<0.05*	0.48±0.43
Within the group	p<0.001*		p<0.05*
Month 3	0.35±0.21	N.S	0.34±0.30
GI			
Baseline	0.73±0.44	p<0.05*	0.49±0.45
Within the group	p<0.001*		p<0.05*
Month 3	0.42±0.38	p<0.05*	0.26±0.25
PD (mm)			
Baseline	2.18±0.72	p<0.05*	1.81±0.66
Within the group	N.S		N.S
Month 3	1.91±0.55	N.S	1.66±0.54
CAL (mm)			
Baseline	2.22±0.77	p<0.05*	1.90±0.75
Within the group	N.S		N.S
Month 3	1.94±0.59	N.S	1.71±0.61
BOP (%)			
Baseline	25.10±23.10	N.S	17.42±18.95
Within the group	p<0.001*		p<0.001*
Month 3	11.65±11.91	N.S	7.16±9.66
TNF-alpha (pg)			
Baseline	8.42±7.4	N.S	9.66±1.6
Within the group	N.S		N.S
Month 3	6.56±5.7	N.S	9.06±1.3
IL-6 (pg)			
Baseline	2.87±3.8	0.001*	0.81±1,6
Within the group	N.S		N.S
Month 3	1.45±2	N.S	0.73±1.7
Visfatin (pg)			
Baseline	11.71±7.45	N.S	8.81±4.8
Within the group	p<0.05*		N.S
Month 3	6.83±3.8	N.S	7.44±5.2

^a^Comparison of weight, height, BMI, WC, total cholesterol, triglyceride, HDL, LDL, PI, GI, PD, CAL, BOP, TNF-alpha, IL-6, visfatin values between O and nO groups by Mann Whitney-U test

*p<0.05, Mann Whitney-U test

BMI: Body mass index; WC: Waist circumference; HDL: High density lipoprotein; LDL: Low density lipoprotein; GI: Gingival index; PD: probing depth; CAL: Clinical attachment level; BOP: Bleeding on probing; TNF-alpha: Tumor necrosis factor-alpha; IL-6: interleukin-6; N.S: Non significant

### Comparison of data among O-Ctrl, nO-CP, O-Ctrl, and O-CP groups

The demographic values of the study population are given in Table 3. There were significant differences in the gender and age of individuals between groups (*P*<0.05). Most (90%) of the O-Ctrl group, 100% of the O-CP group, and 80% of the nO-Ctrl group were female, whereas only 22.2% of the nO-CP group was female. The O-Ctrl group was younger than the other groups. There were statistically significant differences between groups in terms of tooth brushing frequency and interproximal cleaning (*p*<0.05). Half (50%) of the O-Ctrl group, 66.7% of the O-CP group, and 77.8% of the nO-CP group were brushing their teeth once per day, whereas all individuals from the nO-Ctrl group were brushing their teeth twice per day. Most (90%) of the O-Ctrl, O-CP, and nO-CP groups did not care about interproximal cleaning, whereas 50% of the nO-Ctrl group was performing interproximal cleaning regularly (Table 3). As expected, at baseline and at the third month, patients from the O-Ctrl and O-CP groups had higher weight, BMI and WC values compared to their non-obese peers (*p*<0.05). Initial total cholesterol, HDL, LDL, and triglyceride values were similar between groups (*p*>0.05). During the follow-up period, we detected no statistically significant changes in lipid profiles (Table 4). The PI, GI, PD, CAL and BOP scores of O-CP and the nO-CP group were higher compared to both O-Ctrl and nO-Ctrl groups at baseline. These differences were not statistically significant. In the O-CP and nO-CP groups, compared to baseline, the mean values of all periodontal parameters were significantly lower after both periodontal and metabolic diet control treatments (*p*<0.05). Only BOP scores were statistically changed in O-Ctrl and nO-Ctrl groups during the study period (*p*<0.05) (Table 4). At baseline, compared to the nO-Ctrl group, IL-6 levels were higher in both of the obese groups (*p*<0.05), whereas TNF-α values were similar among groups. No significant changes were detected in the GCF profiles of TNF-α and IL-6 in all groups during the study period (*p*>0.05). The total amount of visfatin was greater in patients with O-CP than in the other groups (*p*<0.001). As predicted, following treatment, a significant decrease in the GCF levels of visfatin was observed in both the O-Ctrl and O-CP groups (*p*<0.05) (Table 4).

**Table 3:** Demographic characteristics of obese patients with chronic periodontitis (O-CP), obese individuals without chronic periodontitis (O-Ctrl), non-obese patients with chronic periodontitis (nO-CP) and non-obese individuals without chronic periodontitis (nO-Ctrl)

	O-Ctrl	O-CP	nO-Ctrl	nO-CP	P value^**a**^
	**(n=10)**	**(n=21)**	**(n=10)**	**(n=9)**	
Age (years) (mean±SD)	46.50±12.0	44.67±10.87	27.80±3.12	44.22±6.74	
Female/male (n)	9/1	21/0	8/2	2/7	0.001*
Smoking (yes/no) (n)	2/8	5/16	1/9	4/5	0.373
Frequency of toothbrushing (n) (%)					0.002*
Once a day	5	14	0	7	
Twice a day	4	5	10	2	
Seldom	1	2	0	0	
Interdental cleaning (n) (%)					0.001*
Regular	0	0	5	0	
Rare	1	0	1	0	
None	9	21	4	9	

^a^Comparison of age, female/male, smoking, frequency of toothbrushing and interdental cleaning percentages between O-CP, O-Ctrl, nO-CP and nO-Ctrl groups by Chi-square test, Fischer’s exact test

*P<0.05, Chi-square test, Fischer’s exact test

**Table 4:** Anthropometric, laboratory, periodontal parameters and total amounts of adipocytokines in GCF for O-CP, O-Ctrl, nO-CP and nO-Ctrl Groups

		Baseline		3 Months			

	n	Mean±SD	Between Groups P values^**a**^	Mean±SD	Between Groups P values^**a**^	Within Groups	
Weight (kg)	O-Ctrl	10	93.10±15.01	0.001*	91.44±15.41	0.001* 0.008*	N.S
	O-CP	21	89.72±12.69	1-3	88.32±12.20	1-3 1-4	N.S
	nO-Ctrl	10	59.90±8.92	2-3	59.90±8.92	2-3 2-4	N.S
	nO-CP	9	75.00±8.75		74.56±8.59		N.S
Height (m)	O-Ctrl	10	162,70±9,06	0.007*	162.70±9.06	0.007*	N.S
	O-CP	21	161,14±5,98	2-3	161.14±5.98	2-3	N.S
	nO-Ctrl	10	169,67±5,22		169.67±5.22		N.S
	nO-CP	9	166,10±7,96		166.10±7.96		N.S
BMI (kg/m2)	O-Ctrl	10	35.07±5.03	0.001*	34.30±4.96	0.001*	N.S
				1-4		1-4	
	O-CP	21	34.61±5.90	2-4	33.93±5.65	2-4	N.S
	nO-Ctrl	10	21.40±1.33	1-3	21.40±1.33	1-3	N.S
	nO-CP	9	25.89±1.87	2-3	25.72±1.86	2-3	N.S
WC (cm)	O-Ctrl	10	107.10±10.14	0.001*	106.00±9.91	0.001*	N.S
				1-4		1-4	
	O-CP	21	105.19±10.97	2-4	104.00±10.57	2-4	N.S
	nO-Ctrl	10	71.30±7.99	1-3	71.30±7.99	1-3	N.S
	nO-CP	9	85.89±12.40	2-3	85.89±12.40	2-3	N.S
Total Cholesterol (mg/dl)	O-Ctrl	10	197.10±27.61	N.S	184.30±40.21	N.S	N.S
	O-CP	21	208.00±34.62		200.33±38.32		N.S
	nO-Ctrl	10	177.78±32.18		177.78±32.18		N.S
	nO-CP	9	190.10±24.53		181.44±39.87		N.S
Triglyceride	O-Ctrl	10	128.70±50.67	N.S	117.40±43.68	N.S	N.S
(mg/dl)	O-CP	21	136.05±46.17		138.52±51.64		N.S
	nO-Ctrl	10	109.89±43.01		109.89±43.01		N.S
	nO-CP	9	111.40±77.14		113.44±39.21		N.S
HDL (mg/dl)	O-Ctrl	10	55.10±17.20	N.S	50.90±16.51	N.S	N.S
	O-CP	21	50.57±9.52		55.14±13.28		N.S
	nO-Ctrl	10	47.89±15.30		47.89±15.30		N.S
	nO-CP	9	62.10±12.74		49.67±16.66		N.S
LDL (mg/dl)	O-Ctrl	10	116.20±23.15	N.S	111.60±34.88	N.S	N.S
	O-CP	21	128.64±28.50		116.14±38.74		N.S
	nO-Ctrl	10	107.44±30.29		107.44±30.29		N.S
	nO-CP	9	112.50±28.31		113.33±39.69		N.S
PI	O-Ctrl	10	0.39±0.28	0.001*	0.19±0.05	0.001*	
	O-CP	21	1.01±0.46	1-2	0.43±0.22	1-2	0.05*
	nO-Ctrl	10	0.15±0.13	2-3	0.24±0.33	1-4	
	nO-CP	9	0.85±0.34	4-3	0.47±0.22	3-4	0.05*
GI	O-Ctrl	10	0.21±0.11	0.001*	0.14±0.07	0.001*	
				1-2		1-2	
	O-CP	21	0.98±0.28	2-3	0.56±0.40	2-3	0.05*
	nO-Ctrl	10	0.12±0.12	1-4	0.10±0,09	1-4	
	nO-CP	9	0.89±0.30	3-4	0.43±0.27	3-4	0.05*
PD (mm)	O-Ctrl	10	1.32±0.21	0.001*	1,31±0,25	0.001*	N.S
				1-2		1-2	
	O-CP	21	2.59±0.46	2-3	2,19±0,41	2-3	N.S
	nO-Ctrl	10	1.26±0.32	1-4	1,22±0,31	1-4	N.S
	nO-CP	9	2.43±0.21	3-4	2,13±0,26	3-4	N.S
CAL (mm)	O-Ctrl	10	1.32±0.21	0.001*	1,31±0,25	0.001*	N.S
				1-2		1-2	
	O-CP	21	2.65±0.53	2-3	2,24±0,45	2-3	N.S
	nO-Ctrl	10	1.26±0.32	1-4	1,22±0,31	1-4	N.S
	nO-CP	9	2.60±0.31	3-4	2,25±0,32	3-4	N.S
BOP (%)	O-Ctrl	10	5.40±3.75	0.001*	0,40±1,26	0.001*	0.002*
				1-2		1-2	
	O-CP	21	34.48±22.53	2-3	17,19±10,62	2-3	0.002*
	nO-Ctrl	10	1.20±3.79	1-4	0,00±0.00	1-4	N.S
	nO-CP	9	35.44±9.89	3-4	14,67±9,37	3-4	0.002*
TNF-alpha (pg)	O-Ctrl	10	7.21±9.77	N.S	5.22±5.42	N.S	N.S
	O-CP	21	9.00±6.10		7.20±5.91		N.S
	nO-Ctrl	10	8.86±0.87		7.18±5.20		N.S
	nO-CP	9	10.54±1.80		9.30±1.29		N.S
IL-6 (pg)	O-Ctrl	10	1.32±0.84	0.001*	0.81±0.72	N.S	N.S
	O-CP	21	3.61±4.43	1-3	1.76±2.29		N.S
	nO-Ctrl	10	0.00±0	2-3	0.00±0		N.S
	nO-CP	9	1.71±2.01		1.56±2.23		N.S
Visfatin (pg)	O-Ctrl	10	11.62±10.71	0.001*	6.65±4.08	N.S	0.048*
	O-CP	21	21.53±39.55	1-2	6.96±3.49		0.039*
	nO-Ctrl	10	7.15±3.12	2-3	7.14±3.12		N.S
	nO-CP	9	10.65±5.72	2-4	7.43±5.20		N.S

^a^Comparison of weight, height, BMI, WC, total cholesterol, triglyceride, HDL, LDL, PI, GI, PD, CAL, BOP, TNF-alpha, IL-6, visfatin values between O-CP, O-Ctrl, nO-CP and nO-Ctrl groups by Kruskal-Wallis

*P<0.05, Kruskal-Wallis

### Correlations

At the beginning of the third month, we detected a positive correlation between changes in weight and PI values in the O-CP group (*p*<0.05). During the follow-up period, there was a positive correlation between the change in LDL levels and the change of BOP scores in the O-CP group (*p*<0.05). BMI and WC were only positively correlated with GI scores for the O-Ctrl group (*p*<0.05).

## Discussion

To the best of our knowledge, this study is the first of its kind to analyze the total amount of GCF visfatin levels as a possible biomarker in obese individuals with or without periodontal disease. All individuals with periodontitis had GCP and obesity based on BMI and WC. Overall, our findings demonstrated that the total amounts of GCF, visfatin, and IL-6 were higher in obese individuals compared to their non-obese controls and in individuals with GCP compared to their healthy periodontal controls. Additionally, by the end of the observation period, visfatin levels had decreased (*vs*. baseline) in the obese groups. These results indicate that obesity and periodontitis can, independently or together, change the levels of pro-inflammatory adipocytokines in GCF.

Fat levels are dynamic over a lifetime and vary according to age, gender, ethnicity, level of physical activity, and hormonal status. The proportion of females was greater in the O group (98%) than in the nO group (52.6%). Dalla-Vecchia, et al.^10^(2005) reported that women have a higher prevalence of body fat than men, regardless of age, from puberty onwards. Nevertheless, Saxlin, et al.^11^ (2011) suggest that gender does not influence on the association between obesity and periodontitis. Based on the current study design, it is not possible to explain the influence of gender on the association between obesity and the development of periodontitis. We suggest that biological and hormonal changes may play a role in this difference between genders. It is known that female patients are influenced by hormonal changes in the process from puberty to menopause. This may be explained by the fact that the female patients of this study have more than one pregnancy, their daily activities are limited and they are not active in their working life. One of the other reasons is that many of our socio-cultural traditions are easily able to over-eat high-calorie foods and promote more carbohydrate intake. The fact that the total cholesterol, HDL, LDL, and triglyceride values we obtained in our study did not differ between the groups supports this view. With age, the percentage of body fat increases for both men and women. In addition, the obese patients were older than the non-obese participants in this study. As mentioned earlier, obesity and aging are associated with periodontitis.^12^ One possible explanation for these results is obesity; aging is consistently associated with low-grade inflammation. Other hypotheses are the prevalence of periodontitis in older individuals, prolonged exposure to periodontal bacteria, age-related changes in immune response, or chronic systemic disease. The frequency of tooth brushing and interdental cleaning was lower in the O group than in the nO group. Consequently, we found that initial PI and GI measurements were significantly higher in the obese group compared to the nO group. This finding might be due to insufficient oral hygiene and eating habits.

Comparing baseline and post-treatment values, we detected no significant changes in weight, BMI or waist circumference in either of the obese groups. These results suggest that changes to the diets of obese individuals are not sufficient to produce weight loss and, at the same time, increase their physical activity. The clinical parameters of periodontal disease showed a considerable increase in patients with obesity compared to non-obese patients. Studies have investigated the relationship between BMI and periodontal parameters,^13-17^ some of which have reported that the measurements of periodontal indices were higher in obese than non-obese subjects.^13,15,16^ In the present study, PI, GI, PD, CAL, and BOP measurements were significantly higher in the O-CP and nO-CP groups than in the O-Ctrl and nO-Ctrl groups. Periodontal parameters, with the exception of BOP, were higher in the O-CP group than in the nO-CP group. However, no statistically significant differences were observed. Similar findings were also made when comparing the O-Ctrl and nO-Ctrl groups. We also detected a significant decrease PI, GI, and BOP following the instruction of oral hygiene and changing of eating habits in the O-CP and nO-CP groups. Similarly, Kongstad, et al.^15^(2009) found a positive relationship between obesity and BOP. Additionally, Altay, et al.^16^ (2013) demonstrated that obesity increased PI compared to non-obese subjects, but there was no significant difference in GI, PD, and BOP values.^16^ In contrast, Gursoy, et al.^14^ (2006) and Zuza, et al.^17^(2011) have indicated that obesity does not affect periodontal parameters. Obese individuals generally ingest large quantities of calories from foods with saturated fat, sugary food, and foods of low nutritional value, which appears to contribute to poor oral health. After diet modification, intake of fibrous foods increased, and consumption of fatty and sugary foods decreased. The psychological adaptation to the new diet program and lifestyle might partially contribute to the improvement of periodontal conditions, as can be seen in our study in an obese group with GCP. The main driver for this improvement in periodontal parameters might be the instructions addressing oral hygiene habits.

The biological mechanisms underlying the relationship between obesity and periodontal diseases have not been fully elucidated, but adipose tissue-derived cytokines might have a key role.^9^ Adipocytes cause hyperinflammatory responses by over expressing adipokines and adipocytokines^9^. Accordingly, it has been proposed that the increased amount of adipose tissue in obese individuals triggers the development of periodontitis.

Visfatin is thought to have insulin-like effects, particularly those that lower plasma glucose levels.^7,18^ Also, it has been suggested that visfatin release in fat cells is independently associated with obesity and metabolic syndrome.^8,18^ The abundance of adipose tissue in these patients is responsible for elevated levels of visfatin.^18,19^ In our study, the mean GCF visfatin level was 11.71 pg in the obese group and 8.81 pg in the nO group. However, this difference was not statistically significant. The authors reported that the level of visfatin was higher in obese women than in non-obese participants.^20^ The same study also reported a correlation with plasma visfatin and body weight reduction using an exercise program.^20^ Similarly, in the present study, the total amount of visfatin in GCF was significantly increased in patients with O-CP compared to the other groups. As predicted, significant decreases in GCF levels of visfatin were observed in both the O-Ctrl and O-CP groups throughout the study period. The present study findings are in line with recent studies.^7,8,21^ Pradeep, et al.^8^(2011) evaluated the relationship between the serum and GCF concentrations of visfatin and periodontal diseases, concluding that visfatin concentration increased with disease severity in the serum and GCF. In addition, the concentration of visfatin was higher in patients with GCP and type 2 diabetes mellitus (T2DM) compared to systemically healthy individuals with periodontal disease.^8^ Mohamed, et al.^22^ (2015) reported a positive correlation between PD and visfatin levels in GCF, and they found that visfatin increased significantly the number of diseased sites. Türer, et al.^23^ (2015) showed that GCF and serum visfatin levels are higher in patients with chronic periodontitis than those with gingivitis and healthy controls. They also reported that visfatin levels were significantly decreased following periodontal treatment. Ghallab, et al.^21^(2015) detected significantly higher visfatin mRNA and protein expression in patients with CP+T2DM compared to controls, which is in agreement with a previous study. It has been reported that salivary visfatin levels increase in patients with periodontal disease and that there is a relationship between salivary visfatin concentration and periodontal infection.^24^ A recent study reported significantly higher expression of visfatin, NF-κB, PI3k, TNF-α, and IL-1β in gingivitis and periodontitis groups compared to healthy individuals.^25^ These authors suggest that increased visfatin levels play a role in the pathogenesis of periodontitis.

TNF-α and IL-6, which are known as tissue destruction mediators, function as pro-inflammatory cytokines, and their levels increase during inflammation.^26^ In GCP, these cytokines are thought to play an important role on the pathogenesis of the disease by stimulating bone resorption.^26^ In patients with severe periodontitis, corpuscles and adipocytes in the liver have been proposed as IL-6 sources.^2,3^ This information helps to understand the high level of GCF IL-6 in obese individuals. Previous studies have demonstrated that the systemic level of IL-6 and TNF-α increase in obese individuals and decrease after weight loss.^2,3,17^ A significant relationship between serum IL-6 and BMI and WC have been reported, in the absence of a relationship with TNF-α.^27^ Recent studies reported both higher levels of IL-6 and TNF-α in overweight and obese individuals with periodontitis compared to non-obese counterparts.^28,29^ Khaodhiar, et al.^30^(2004) also found a significant association between obesity and IL-6 in their study of obese subjects, but not with TNF-α. Similarly, when we investigated the initial levels of IL-6 and TNF-α in our study, IL-6 levels were higher in the obese group compared to the non-obese group (*p*<0.001), whereas TNF-α and visfatin values were similar in both groups. Additionally, the baseline IL-6 values of the O-CP group of patients were statistically higher in the nO-Ctrl group. At the same time, the fact that IL-6 is significantly higher in the O-Ctrl group than in the nO-Ctrl group suggests that obesity alone can trigger the level of inflammation seen in GCF. Possible reasons for the different findings of these studies include different study designs and use of different statistical methods. The relationship between obesity, periodontal status and serum IL-6 remains controversial due to the role of TNF-α. Possible explanations for increased GCF levels of IL-6 might be that macrophages have a greater infiltration of both systemic and local circulation in obese individuals, and might lead to increased expression of adipokines, compared to normal weight individuals.

This study has some limitations. First, in calculating the sample size, the low number of participants should be sufficient to reach significant results, but it is not possible to define clinically important differences in all parameters. Therefore, multicenter studies with larger populations are needed to validate the present study findings. Secondly, most obese participants included in this clinical trial were moderately obese. In addition, depth of PD and CAL were not categorized due to of sample size. Future interventional studies are needed to define the levels of adipocytokines in more severe cases of obesity (obese class-III), including those with and without periodontitis, and categorized according to PD and CAL depth (e.g., shallow, mild to moderate, and deep).

## Conclusion

In conclusion, the results of this study suggest that visfatin and IL-6 levels might play a role in the pathogenesis of obesity and periodontal disease and that these can be used as reliable markers for monitoring the progress of both diseases. Moreover, our findings demonstrate that, in addition to obesity, lipid profiles can be associated with periodontitis in systemically healthy people.
